# The games economists play: Why economics students behave more selfishly than other students

**DOI:** 10.1371/journal.pone.0183814

**Published:** 2017-09-05

**Authors:** Philipp Gerlach

**Affiliations:** Max Planck Institute for Human Development, Berlin, Germany; Middlesex University, UNITED KINGDOM

## Abstract

Do economics students behave more selfishly than other students? Experiments involving monetary allocations suggest so. This article investigates the underlying motives for the economic students’ more selfish behavior by separating three potential explanatory mechanisms: economics students are less concerned with fairness when making allocation decisions; have a different notion of what is fair in allocations; or are more skeptical about other people’s allocations, which in turn makes them less willing to comply with a shared fairness norm. The three mechanisms were tested by inviting students from various disciplines to participate in a relatively novel experimental game and asking all participants to give reasons for their choices. Compared with students of other disciplines, economics students were about equally likely to mention fairness in their comments; had a similar notion of what was fair in the situation; however, they expected lower offers, made lower offers, and were less willing to enforce compliance with a fair allocation at a cost to themselves. The economics students’ lower expectations mediated their allocation decisions, suggesting that economics students behaved more selfishly because they expected others not to comply with the shared fairness norm.

## Introduction

Economists seem to have never enjoyed a good reputation among their peers. In 1849, historian Thomas Carlyle described economics as “the dismal science” [1]. Thirty years later, economist Francis Walker felt compelled to explain why economists “tend to be in bad odor amongst real people” [2]. And in 1945, psychoanalyst Donald Winnicott denounced economics as the “science of Greed” [3]. More recently, rather than working in the realm of speculation, researchers have sought to determine whether there is a sound empirical basis for economists’ bad reputation. In particular, the results of experiments showing links between economic training and more selfish choices have lent firm support to the critics of the discipline: Economics students have been found to behave more selfishly than other populations across various situations involving monetary allocations [4–15].

Yet selfish behavior—like all behavior—is not free from context and may be driven by various motives. This article aims to shed a light on the potential links between studying economics and selfish behavior. More precisely, it investigates *why* economics students behave more selfishly than other people do. To this end, the article distinguishes three theoretical mechanisms that may account for more selfish behavior in economics students: economics students are less concerned with fairness when making allocation decisions; they are equally concerned with fairness but have a different notion of what is fair; they expect others to behave more selfishly and therefore feel less obliged to behave fairly themselves. These three mechanisms are empirically tested by comparing the decisions of students from various disciplines in a third-party punishment game. The results suggest that, relative to their fellow students, economics students are about equally likely to be concerned with fairness when making decisions; they have a similar notion of what is fair; but their greater skepticism about others’ behavior mediates their more selfish behavior.

This article is organized as follows: The subsequent section outlines findings that suggest economics students behave more selfishly than others. Then, the three theoretical mechanisms potentially underlying this pattern of results are presented. Next, the experimental design is described, followed by the experimental results. Finally, the design, the results and the potential reasons for economics students’ greater skepticism are discussed.

## Experiments and economics students

A central finding of experiments involving monetary allocation decisions is that substantial numbers of people do not behave according to the predictions of game theory [16,17]. Participants in such experimental games are frequently willing to contribute to the other participant’s welfare in a non-trivial fashion—even at their own expense [18–21]. In public goods games, for instance, participants can choose how much of their private savings to contribute to a common pot, which is then multiplied and evenly distributed among all participants [22]. The configuration of public goods games is such that participants are tempted to save everything privately and to contribute nothing to the common pot. This “free riding” is what game theory predicts for participants whose goal is to maximize payment. Yet, the less participants free ride, the less everybody earns—the “tragedy of the commons” [23]. Despite this bleak prediction findings from Western countries show that participants regularly contribute 40% to 60% of their initial stocks [24,25]. There is, however, at least one systematic exception to this finding.

In a series of experiments, Marwell and Ames [26] discovered that first semester economics graduate students contributed on average only 20% of their private savings to the common pot. In other words, the choices of the economics students were inclined towards free riding and thus consistent with the predictions of game theory. Despite some limitations in the experiments—for example, the samples were not strictly comparable—and a failed replication attempt [27], the work by Marwell and Ames stimulated a number of follow-up studies on whether economics students behave more selfishly than their peers. Overall, the investigations largely supported this claim: Economics students offered and accepted smaller amounts in ultimatum games [4]; defected more in prisoner’s dilemmas [5,6]; deceived more in cheap talk games [7,8]; contributed less in threshold public goods games [9]; gave less in “solidarity games” [10]; shared less in dictator games [11–13]; trusted and reciprocated less in trust games [14]; and were less prosocial and more competitive in decomposed games [15].

Three theoretical mechanisms have been proposed to explain the more selfish behavior of economics students. The first argues that—in contrast to other students, who often indicate fairness as a motive driving their choices [28,29]—economics students are less concerned about fairness when making their decisions. The seminal investigation of Marwell and Ames [26], for example, found that “the economics graduate students were about half as likely as other subjects to indicate that they were ‘concerned with fairness’ in making their investment decision” [11,26]. This would suggest that economics students’ behaviors are driven by motives other than fairness.

Marwell and Ames also speculated about an alternative theoretical account for their observation. Overall, they found “surprising unanimity of thought regarding what was considered fair” among the participants [26]. Yet comparing economics students with other participants proved difficult because

[m]ore than one-third of the economists either refused to answer the question regarding what is fair, or gave very complex, uncodable responses. It seems that the meaning of “fairness” in this context was somewhat alien for this group. Those who did respond were much more likely to say that little or no contribution was “fair” [26].

This explanation differs from the first: it suggests that economics students may have been as concerned about fairness as other students, but that they had a different notion of what was fair [11].

A third theoretical mechanism suggests that economics students behave more selfishly due to their greater skepticism about the fair behavior of other people [8,30,31]. This mechanism is derived from more general theorizing on social norms [32–35]. According to social norms theories, people first define a social situation as an exemplar of a class of social situations with which they are familiar (e.g., this situation resembles situations of class A). They then associate behavioral rules with that class of social situations (e.g., in situations of class A the rule is to split the endowment about equally). The underlying assumption is that people prefer to comply with the associated rule if two conditions are fulfilled: The individual expects (1) that rule following is what is the normative standard (e.g., what is fair) and (2) other people follow the rule as well [36,37]. The hallmark of social norms is that people are willing to sanction rule deviant behavior of others—even that of third parties—at a cost to themselves [38]. Extrapolated to selfish behavior, economics students may thus share the motives and the notion of fairness, yet they simply do not expect others to behave fair [30,31]. This skepticism can give the impression that selfishness is justified or even desirable [39–41]. As a consequence, the economics students’ greater skepticism would make them feel less obliged to behave fairly themselves and less willing to sanction the norm deviant behavior of others. Overall, the theoretical mechanism of the social norms hypothesis thus assumes that economics students have similar normative standards. However, in contrast to other students economics students are more skeptical. This skepticism is reflected in more selfish choices and a decreased willingness to sanction the unfair behavior of others. It is worth noticing that the social norms hypothesis assumes that economics students other students are equally motivated to comply with social norms. I return to this point in the discussion section.

To the best of my knowledge, no study dissected and measured the relative effect of the three mechanisms to explain why economics students behave more selfishly than other students do. The aim of this study is to fill that gap by means of an experimental game that was likely unfamiliar to all participants at the time the experiment was conducted. The following hypotheses are tested:

**Hypothesis 1**: Relative to other students, economics students are less concerned with fairness when making decisions and therefore behave more selfishly.
**Hypothesis 2**: Relative to other students, economics students have a different notion of fairness and therefore behave more selfishly.
**Hypothesis 3**: Relative to other students, economics students expect other participants to make more selfish decisions and therefore behave more selfishly. The skepticism also makes them less willing to sanction the norm deviant behavior of others.

## Methods

### Participants

The study was conducted at a major British university at the end of the academic year. Undergraduate students from various semesters were recruited via the university’s weekly bulletins. Altogether, 176 students participated in the online study. Eleven participants had to be excluded from the analysis: five participants did not understand the experiment, six did not reveal their field of study. Of the 165 remaining participants, 42 studied economics, 60 studied an art major, 63 studied a science major. The median age was 20 years (*M*_*age*_ = 20.81, *SD*_*age*_ = 3.41) and 104 participants were female. Because the proportion of women studying arts (*M*_*women*_ = 75%) was greater than the proportion of women studying economics (*M*_*women*_ = 55%) and sciences (*M*_*women*_ = 57%) analyses include gender as a predictor. The Research Ethics Committee of the Department of Psychology of the University of Cambridge approved the experiment and its consent procedure. To participate in this study all participants provided their informed consent. The database and the source code can be found in the Supporting Information.

### Materials and procedure

The study involved a third-party punishment game [42,43]. The game consisted of two stages and three roles, which were labeled A, B, and C to avoid evoking specific behaviors ([44]; see Supporting Information for full instructions). For simplicity’s sake, in the following, the roles are referred to as proposer, receiver, and judge, respectively. All participants rotated through all three roles in a fixed order. Decisions were made sequentially and the situation did not repeat itself. About one month after the experiment, the decisions of seven randomly picked groups (21 participants) were matched and the participants were paid in accordance with the outcome of the experiment. The time delay was necessary to complete data collection before matching. All participants were anonymous and fully informed about all aspects of the experiment. There was no deception involved. The average payment was £6.33 among the disbursed participants (about $9.50).

**Fig 1:**
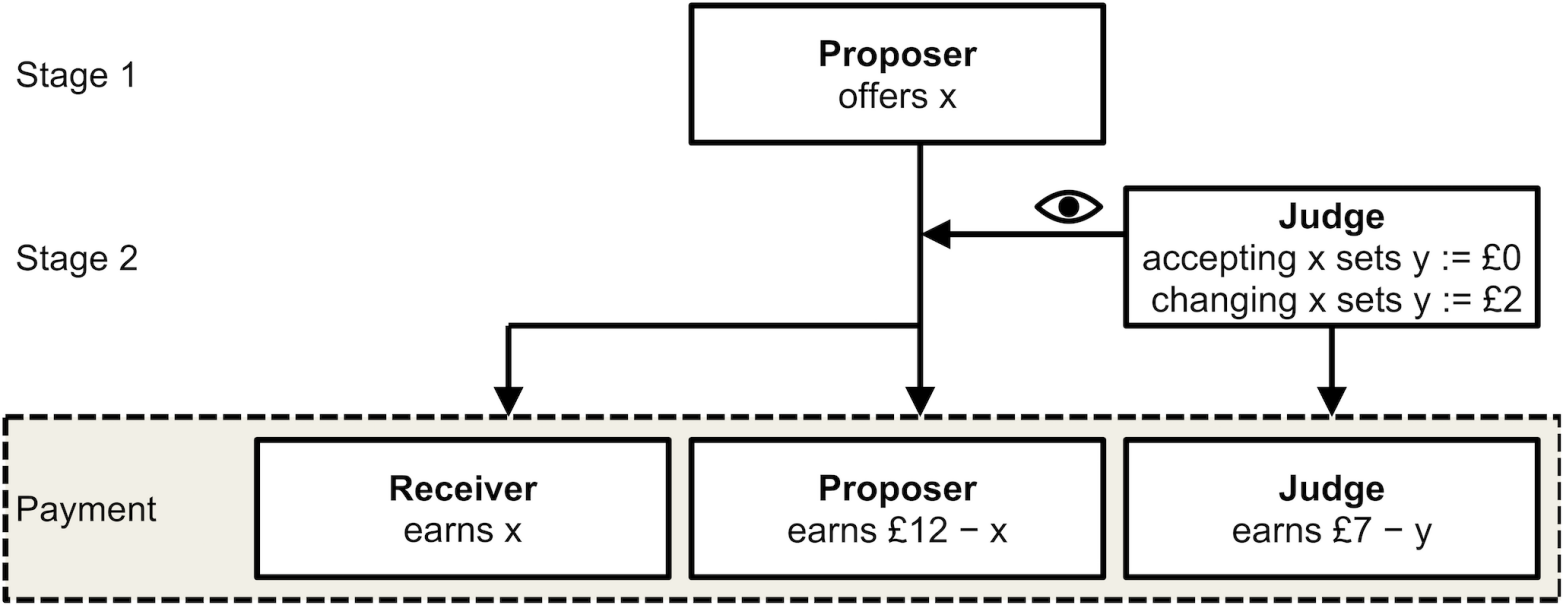
Configuration of the third-party punishment game.

Fig 1 illustrates the two stages of the third-party punishment game. In stage one, proposers were the sole decision-makers. Receivers and judges remained passive. Proposers were endowed with £12 and could offer the receiver any amount between £0 and £12 in whole pounds sterling while themselves keeping the remainder. If, for example, a proposer offered a receiver £5, the proposer’s income at the end of stage one was £7 and the receiver’s was £5. If the proposer offered £0, the proposer’s income at the end of stage one was £12 and the receiver’s was £0. Stage one is thus similar to a dictator game [45], the most common measure of non-selfish motives. Studies suggest that offers in third-party punishment games positively and strongly correlate with donations in dictator games [46].

In stage two, judges were the sole decision-makers. Proposers and receivers were passive. Judges could either accept or veto the proposer’s offer. If judges accepted the offer, proposers and receivers were paid in accordance with stage one. The judges earned £7 and the transaction was complete. If, however, judges vetoed the offer, their earnings were reduced to £5, and they proceeded to re-allocate the £12 between proposer and receiver. For example, in stage one, a proposer might have offered £0. In stage two, the judge vetoed the offer and changed it to £9. In this case, the proposer’s income was £3 (= £12 –£9), the receiver’s £9, and the judge’s £5 (= £7 –£2). If the judge instead accepted the offer, the proposer’s income would be £12 (= £12 –£0), the receiver’s £0, and the judge’s £7 (= £7 –£0). The latter outcome is consistent with game theory: Judges are predicted to accept any offers proposed because vetoing does not benefit them personally; on the contrary, it costs them money. Accordingly, proposers are expected to anticipate that judges will accept any offer and consequently to offer receivers £0.

The third-party punishment game involves two incentivized decisions being made by the two active roles: proposers and judges. In each role, selfish motives compete with other potential motives. Judges may decide to veto a proposer’s offer and reallocate the £12. In so doing, they can establish an equal distribution of money or punish the proposer by allocating a larger share to the receiver. Yet this redistribution comes at a financial cost of £2. A judge may therefore be tempted to permit an unfair offer and keep the full £7. Vetoing can never be in a judge’s selfish interests. Nevertheless, judges may find it worth forsaking £2 to re-distribute the £12 and establish a fair outcome. Vetoing thus reflects judges’ willingness to punish others for violating behavioral rules—such as, fairness norms—at a cost to themselves [38,43].

The motives of proposers are less evident. Proposers may be motivated to maximize their own income, to maximize the receiver’s income, to maximize the group’s income, to establish a fair split, and so on. If proposers choose selfishly and strive to maximize their own payments, they must act strategically and be aware of the judges’ power of veto: At what threshold are judges willing to step in? If proposers are primarily interested in a fair distribution, these considerations are less salient; proposers may assume that judges have no reason to veto an offer that is perceived as fair. To provide insights into the proposers’ motives, all participants in this role were asked to comment on their choice: “In two to four sentences, please explain the reason behind your decision.” This question served primarily to identify the aspect of the situation to which the participant paid most attention. It was assumed that an open-ended question was a less leading way of eliciting participants’ motives than a question directly asking whether fairness concerns were involved (as, for example, in [26]).

Two graduate students in psychology categorized comments into two categories. All comments that mentioned fairness as relevant to the decision were classified as reflecting concerns for fairness. Comments that did not mention fairness were classified as *not* reflecting concerns for fairness. The two coders were blind to the participants’ majors and the hypotheses of the study. In cases of disagreement, the coders met to discuss and reconcile discrepancies. The classifications were used to operationalize fairness concerns (Hypothesis 1). After the transaction was completed, another question assessed participants’ notion of fairness: “What would be a fair allocation of the £12 to the receiver?” Participants could reply to the question by stating a number between £0 and £12 or by replying “don’t know” (Hypothesis 2). To operationalize expectations about the other participants’ choices, receivers were asked—prior to making any payoff relevant decision—how much they expected the proposer would offer them. These expectations and the judge’s vetoing behavior served as independent measures for the social norms hypothesis (Hypothesis 3).

The order in which participants rotated through the game was as follows: Participants were first asked about their expectations. Then, on one screen, they decided and explained their offer. Thereupon, participants were confronted with a randomly assigned offer between £0 and £6, which they could veto or accept. Finally, participants were asked about their perception of a fair offer. The fixed order was chosen for three reasons. First, the question “What would be a fair allocation” was asked *after* all decisions had been made to avoid priming fairness. Second, participants were randomly assigned to offers *after* formulating their expectation and *after* deciding upon the offer to avoid anchoring specific values. I also tested for potential spill over effect between the assigned offer and the subsequent fairness estimate. The assigned offer correlated with neither what was perceived as fair (*ρ* = –0.01, *p* = .916; Spearman’s rank correlation) nor with the likelihood of responding “don’t know” (*t* = –0.98, *p* = .337; Welsh test). Third, participants had to formulate expectations before making an offer to properly test the mediation effect of expectations on decisions, as suggested by the social norms hypothesis.

## Results

### Relative to other students, economics students made more selfish decisions

**Fig 2:**
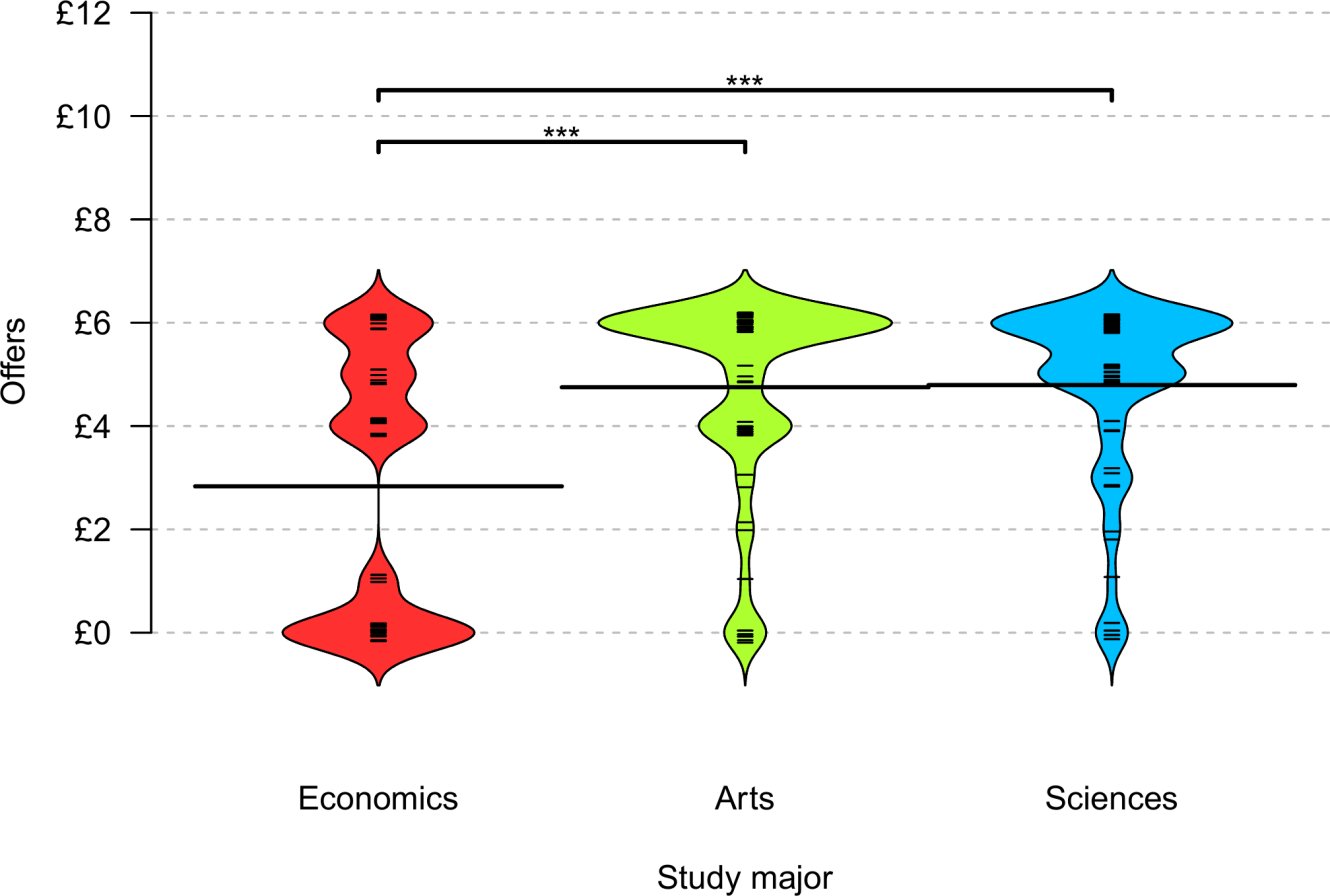
Offers made by study major. The bean plots shows the probability density of the offers per study major, the wider the area the more observation per offer. Each offer is also visualized through a dash. Dashes are vertically jittered for the sake of visualization only. The lower bars indicate the mean offer per major. The upper bars summarize the results of one-sided Wilcoxon rank-sum tests with *** p < .001.

A prerequisite for further analyses was that the third-party punishment game would replicate that economics students behave more selfishly. The offers made by economics students as proposers were indeed the least generous of all student groups. Their average offer (*M* = £2.83, *SD* = 2.56) was about £1.94 smaller than the average offers made by arts majors (*M* = £4.75, *SD* = 2.55; *Z* = 2.47, *p* < .001, *r* = 0.31) and science majors (*M* = £4.79, *SD* = 1.72; *Z* = 4.03, *p* < .001, *r* = 0.31; one-sided Wilcoxon rank-sum tests; arts vs. sciences: *Z* = 0.44, *p* > .21, two-sided Wilcoxon rank-sum tests). Fig 2 illustrates the offers made by study major.

There was also a main effect of gender: male students (*M* = £3.75, *SD* = 2.25) on average made £0.84 smaller offers than female students did (*M* = £4.59, *SD* = 2.09; *Z* = 2.98, *p* = .003, *r* = 0.23, two-sided Wilcoxon rank-sum test). Because gender was unequally distributed across the majors, Table 1 reports the results of a Tobit regression with economics major and gender as predictors. The negative effect of studying economics on the offer made persisted even when gender was controlled for.

**Table 1:** Economics students made lower offers.

Predictor	*b*	*SE*	*z*	*p*
(Intercept)	4.23	0.32	13.17	< .001
Economics	‒2.25	0.42	‒5.41	< .001
Female	0.73	0.37	1.95	.051

*Note*: Tobit regression with offers as the dependent variable. *N* = 165, log-likelihood: ‒344.73, *df* = 326, iterations = 7.

The pattern of economics students behaving more selfishly was thus reproduced in the third-party punishment game. I now turn to the analysis of the three mechanisms potentially underlying this pattern of results.

### Relative to other students, economics students were about as often concerned with fairness when making their decisions

Of the 165 participants, 97 (59%) were classified as mentioning fairness in their comments. For example, one participant offered £6 and commented: “£6 is an equal amount split between the two participants—it seems *fair* that I share the money equally” (emphasis added). Participants mentioning fairness did not necessarily make higher offers, however, as illustrated by this comment on a £3 offer: “This split is deliberately *unfair* in my favor, but not so massively *unfair* that I think C [the judge] would intervene to change the split at the cost of £2 of his own endowment. I would expect C’s threshold to *unfairness* to be higher before he steps in to even” (emphasis added). The remaining 68 participants (41%) did not mention fairness. For example, one participant offered £5 and wrote “C [the judge] can change my decision anyway, and C would like to have 7 pounds instead of 5.” Another participant offered £0, commenting that: “Assuming B [the receiver] is a complete stranger, I owe nothing to B. I have no reason to give any of the £12 to B.”

To rule out that fairness was mentioned only in the context of strategic considerations (as suggested by the second comment), a Fisher’s exact test assessed whether mentioning fairness and mentioning the judge were co-occurring. This was not the case (*p* = .155).

Overall, 48% of the economics students mentioned fairness. Compared to science majors (*M* = 59%, *p* = .319) the proportion of economics students who mentioned fairness was likely the result of sampling error. Compared to arts majors the proportion approached significance (*M* = 67%, *p* = .067; arts vs. sciences: *p* = .456; all Fisher’s exact tests). However, the unequal distribution of gender cofounded the effect (although there was no main effect of gender: males: *M* = 52%; females: *M* = 63%; *p* = .252). To distinguish effects of gender and major, Table 2 presents the results of a binary logistic regression model with major and gender as covariates. Neither major nor gender predicted references to fairness concerns.

**Table 2:** Neither studying economics nor gender predicted references to fairness.

						95% *CI*	
Predictor	*b*	*SE*	*z*	*p*	*OR*	2.5%	97.5%
(Intercept)	0.28	0.28	0.98	.325	1.32	‒0.28	0.83
Economics	‒0.57	0.36	‒1.58	.114	0.56	‒1.29	0.14
Female	0.37	0.33	1.11	.268	1.44	‒0.28	1.01

*Note*: Binary logistic regression with fairness references (yes = 1, no = 0) as the dependent variable. *N* = 165, adjusted *R*^*2*^ = 1%, Nagelkerke’s *R*^*2*^ = 3%, iterations = 4.

### Relative to other students, economics students had a similar notion of fairness in the situation

**Fig 3:**
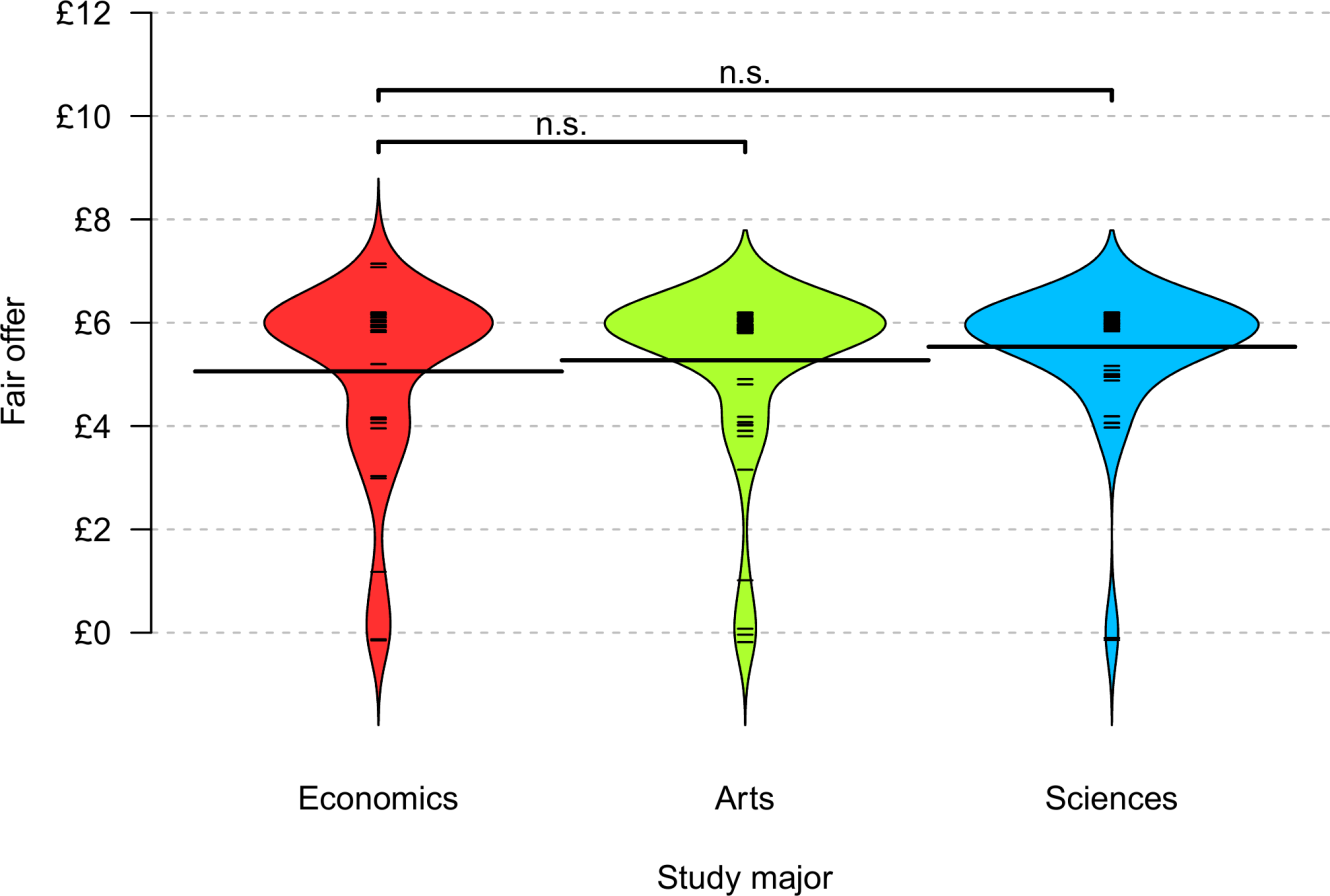
Responses to what would be a fair offer by major. Bean plots and one-sided Wilcoxon rank-sum tests with n.s. = not significant.

After the game ended, participants were asked “What would be a fair allocation of the £12 to role B [the receiver]?” They could state any figure between £0 and £12 or give the response “don’t know.” Fig 3 depicts the findings for those who responded. Students of economics (*n* = 35, *M* = £5.06, *SD* = 1.78), arts majors (*n* = 60, *M* = £5.27, *SD* = 1.43, *p* = .728) and sciences majors (*n* = 58, *M* = £5.32, *SD* = 1.20, *p =* .147) appeared to have similar notions of fairness (all one-sided Wilcoxon rank-sum tests; arts vs. sciences: *p* = .677, two-sided Wilcoxon rank-sum tests).

On average, men stated £0.65 smaller offers to be fair (*n* = 53, *M* = £4.91, *SD* = 1.89) than women did (*n* = 95, *M* = £5.56, *SD* = 1.19, *p* = .008). The Tobit regression model in Table 3 confirmed the gender effect. Yet study major did not predict the offer considered to be fair.

**Table 3:** Studying economics did not predict the notion of fairness but gender did.

Predictor	*b*	*SE*	*z*	*p*
(Intercept)	4.95	0.22	22.23	< .001
Economics	‒0.35	0.30	‒1.17	.244
Female	0.68	0.26	2.59	.010

*Note*: Tobit regression with responses to “what would be a fair allocation” as the dependent variable. *N* = 148, log-likelihood: ‒272.55, *df* = 292, iterations = 5.

Moreover, there were no differences between economics students and other students in the percentage of participants answering “don’t know” (economists: *M* = 17%; arts: *M* = 8%, *p* = .225; sciences: *M* = 8%, *p* = .215; Fisher’s exact tests). Neither was there a gender effect (males: *M* = 13%; females: *M* = 9%; *p* = .429; Table 4).

**Table 4:** Neither studying economics nor gender predicted the response “don’t know.”

						95% *CI*	
Predictor	*b*	*SE*	*z*	*p*	*OR*	2.5%	97.5%
(Intercept)	‒2.18	0.45	‒4.86	< .001	0.11	‒3.06	‒1.30
Economics	0.77	0.53	1.45	.147	2.17	‒0.27	1.82
Female	‒0.39	0.52	‒0.75	.451	0.67	‒1.42	0.63

*Note*: Binary logistic regression with the reply “don’t know” to “what would be a fair allocation”. *N* = 17, adjusted *R*^*2*^ = 1%, Nagelkerke’s *R*^*2*^ = 3%, iterations = 5.

### Relative to other students, economics students expected other participants to make more selfish decisions

**Fig 4:**
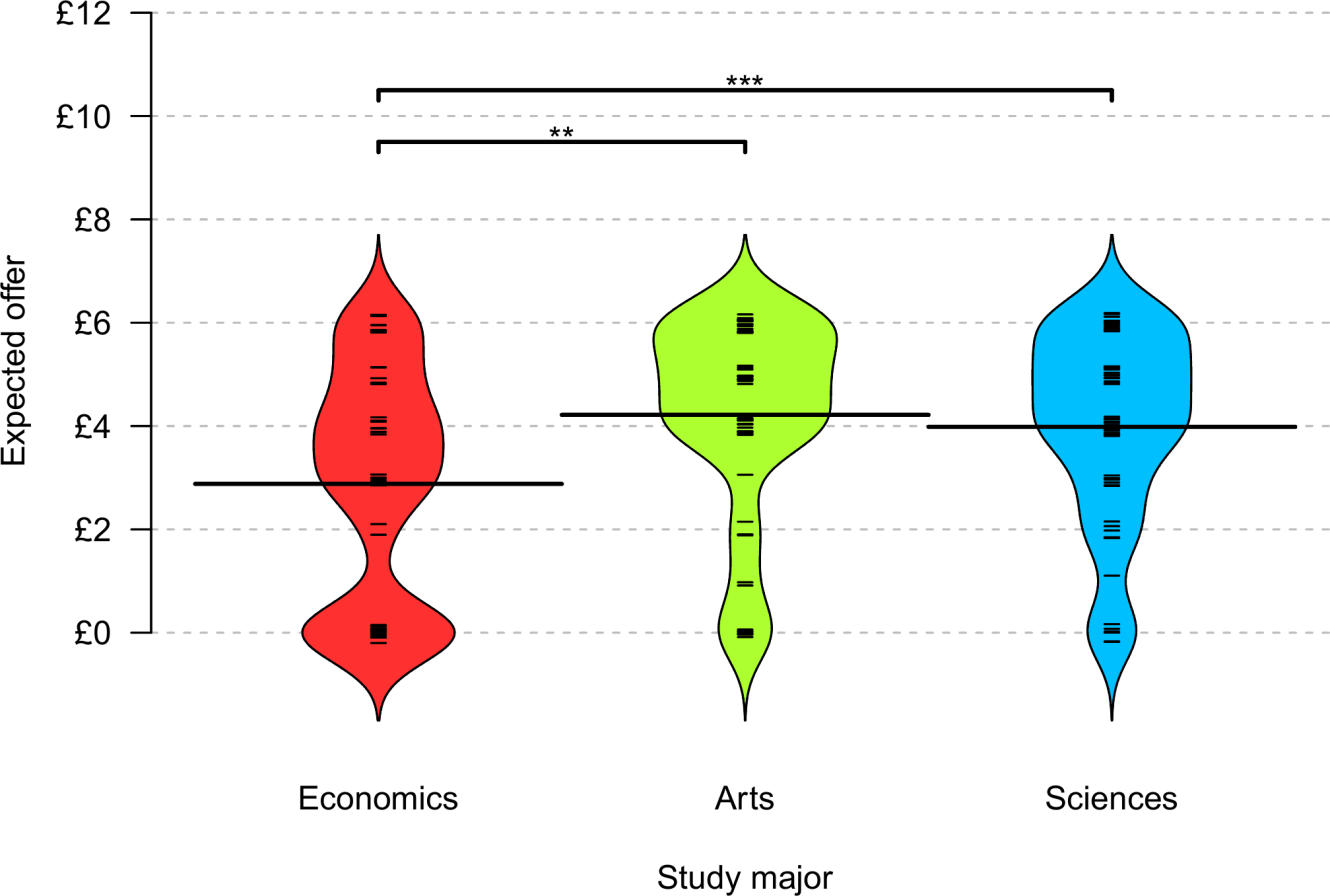
Expected offers by major. Bean plots and one-sided Wilcoxon rank-sum tests with ** p < .01, *** p < .001.

In their role as receivers, all participants were asked how much they expected their assigned proposer to offer. Of all groups, economics students were the most skeptical, expecting on average £1.22 (*M* = £2.88, *SD* = 2.21) smaller offers than arts majors (*M* = £4.21, *SD* = 1.88; *Z* = 2.11, *p* = .001, *r* = 0.25) and science majors did (*M* = £3.98, *SD* = 1.84; *Z* = 2.57, *p* = .005, *r* = 0.32; one-sided Wilcoxon rank-sum tests; arts vs. sciences: p > .342, two-sided Wilcoxon rank-sum tests). Fig 4 illustrates the distributions of expectations across the majors.

There was also a main effect of gender: male students on average expected to be offered £0.77 less (*M* = £3.30, *SD* = 2.05) than female students did (*M* = £4.07, *SD* = 1.95; *Z* = 2.60, *p* = .009, *r* = 0.20, two-sided Wilcoxon rank-sum test). Table 5 presents the results of a Tobit regression model with economics major and gender as predictors. Both covariates predicted the offers expected, with economics major weighing heavier than gender.

**Table 5:** Economics students expected lower offers.

Predictor	*b*	*SE*	*z*	*p*
(Intercept)	3.52	0.31	11.30	< .001
Economics	‒1.39	0.40	‒3.46	< .001
Female	0.75	0.36	2.07	.038

*Note*: Tobit regression with offers as the dependent variable. *N* = 165, Log-likelihood: ‒340.72, *df* = 326, iterations = 6.

Are the smaller offers made by economics students attributable to their lower expectations? To answer this question I built a mediation model, in which a linear regression model for expectations served as a mediator for an “outcome model.” The “outcome model” was similar to the regression model documented in Table 1 but it included expectations as an additional, independent predictor. All models included gender as a covariate. This setup allows to decompose the weights of the dissimilar offers of economics and non-economics students *(τ)* into a mediation effect of expectations *(δ)* and a direct effect as the non-explained remainder *(ζ)* by means of bootstrapping and Monte Carlo simulations with the mediator held constant (for details on causal mediation analysis, see [47]). The proportion mediated can be estimated as the quotient of *δ/τ*. On average, expectations (*δ* = 2.37, *p* < .001) mediated about 53% of the difference between the offers of economics and non-economics students (*τ* = 4.45, *p* < .001; *ζ* = 2.08, *p* < .001).

**Fig 5:**
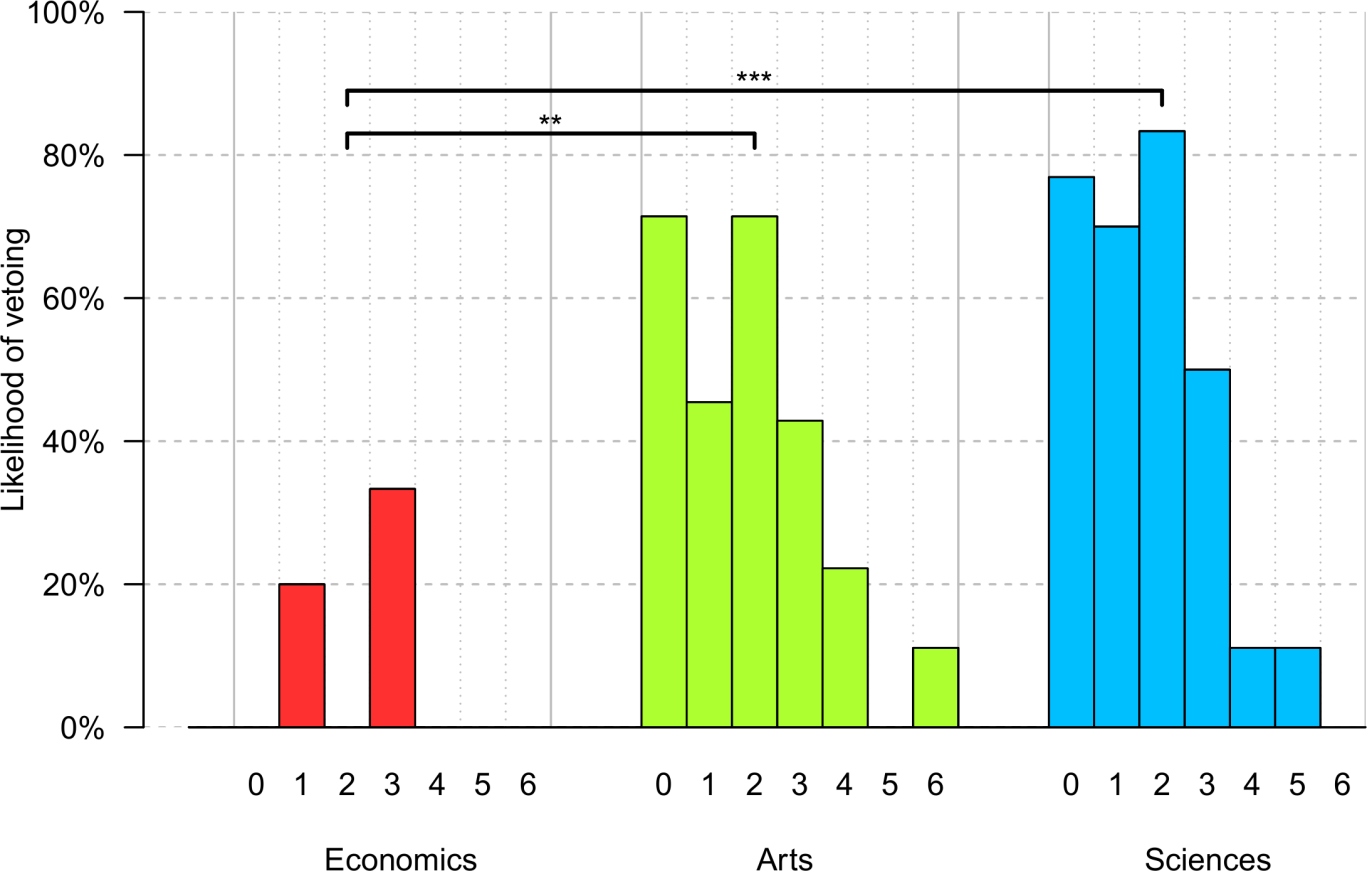
Offers vetoed by study major. Bar plots. The x-axis indicates the observed offer per study major. The y-axis indicates the probability of vetoing as a function of the observed offer (x-axis). Upper bars indicate the results of Fisher’s exact tests for vetoes on offers that were perceived as unfair with ** p < .01, *** p < .001.

Social norms theories also hypothesized that, due to their skepticism of the fair behavior of other people, economics students would be less willing to sanction the rule disconfirm behavior of others. To test this prediction the decisions of the judges were analyzed. For each participant I calculated whether the observed offer was perceived as unfair (i.e., whether the observed offer was less than what was perceived as fair). Among the 119 judges who were presented with offers that they had perceived as unfair economics students (*M* = 16%) were about 3 to 4 times less likely to veto than arts majors (*M* = 48%, *p* = .009, *OR* = 0.21) and sciences majors were, respectively (*M* = 60%, *p* < .001, *OR* = 0.13; arts vs. sciences: *p* = .300, Fisher’s exact tests). Fig 5 plots the likelihood of a veto by the judge’s major as a function of the observed offer.

There was *no* main effect of gender on vetoing (males: *M* = 41%; females: *M* = 49%; *p* = .442, *OR* = 1.36). Table 6 presents the results of a binary logistic regression model with vetoing as the dependent variable. When gender was controlled for, economics students were about 6 (= 1 / 0.16) times less likely to veto unfair offers than other students were.

**Table 6:** Economics students were less likely to veto offers that were perceived as unfair.

						95% CI	
Predictor	*b*	*SE*	*z*	*p*	*OR*	2.5%	97.5%
(Intercept)	1.05	0.47	2.23	.026	2.86	0.13	1.97
Economics	‒1.81	0.63	‒2.88	.004	0.16	‒3.05	‒0.58
Female	0.47	0.46	1.03	.304	1.60	‒0.42	1.36
Observed offer	‒0.53	0.13	‒4.07	< .001	0.59	‒0.74	‒0.28

*Note*: Binary logistic regression with vetoing an offer that perceived as unfair (1 = veto, 0 = accept) as the dependent variable, *N* = 119, adjusted *R*^*2*^ = 42%, Nagelkerke *R*^*2*^ = 33%, iterations = 4.

An overview of the judge’s redistributions is provided in Table 7. The most popular choice (made by 27 of the 56 vetoing players) was an even split of £6 between proposer and receiver.

**Table 7:** Redistribution of vetoed offers.

	Chosen redistribution to receiver											
Assigned offer	4	5	6	7	8	9	10	11	12
0	0	0	3	5	3	0	1	0	3
1	1	1	8^a^	1	0	0	0	2	0
2	1	2	9	1	1	0	1	0	0
3	0	0	4^a^	2^a^	0	1^a^	0	0	1
4	0	1	2	0	0	0	0	0	0
5	0	0	0	1	0	0	0	0	0
6	0	0	1	0	0	0	0	0	0
Total	2	4	27	10	4	1	2	2	4

Note.

^a^ indicates that there was exactly one economics student in the group

## Discussion

This study demonstrated that, relative to their fellow students, economics students offered less in a third-party punishment game; they were about equally likely concerned with fairness, and they had a similar understanding of what was fair. However, economics students expected to receive smaller offers from others, which in turn mediated their own smaller offers. Moreover, economics students were less willing to veto unfair allocation of others. Taken together, the results suggest that economics students’ more selfish behavior is not due different fairness standards but to social norms.

Several limitations to this investigation and the conclusions that may be drawn from it warrant consideration. Foremost, economics students’ expectations only *partly* mediated the offers they made, which suggests that their greater skepticism is not the only explanation for their more selfish behavior. Further, participants may have decided how much to offer *before* forming expectations about others. If this were the case, causality would be reversed, with decisions informing expectations (in the sense “If I do B in the situation of class A, expect B to be the rule for situations of class A”). Reverse causality was addressed primarily through the study design: expectations were measured *before* participants decided on an offer. Nonetheless, it cannot be completely ruled out that participants made a hypothetical decision immediately after reading the instructions [48].

Moreover, offers in the third party punishment game cannot be unequivocally interpret as due to non-selfish motives only. As suggested by some of the participants’ comments, high offers can be due to strategic motives, to avoid vetoing. Although studies suggest that offers in the third party punishment positively and strongly correlate with established measures of selfishness [46] and the proposer’s offers primarily served as a replication of economics students’ greater propensity to behave selfishly, a combination of experimental games would have been preferable to directly test the economics students’ more selfish motives. Such combination of experimental games could also shed light on differences in the motives to veto. In the particular version of the third party punishment game vetoing always caused judges to earn less than what one or both other participants would earn. However, third party punishment in general can be motivated not only by norm enforcement but also by spiteful motives [49]. Further experiments could not only assess the students’ differences in their motives to veto but also test a central assumption of the social norms hypothesis, namely whether economics students and other students are similarly motivated to comply with social norms.

Another limitation is the cross-sectional design of the study. Why are economics students more skeptical in the first place? There are two possible explanations. One is that people who are more skeptical and behave more selfishly are drawn to study economics (self-selection hypothesis). The other explanation is that economics students learn to associate specific situations with more selfish behaviors (socialization hypothesis). It is also possible that both explanations hold. The two effects have been extensively discussed in the literature ([50,51]; see [52] for an overview). In the specific context of experimental games, the findings of cross-sectional studies that correlate academic year with choices are frequently interpreted as pointing to socialization [5,8,14] rather than self-selection [4], suggesting that economics students learn to behave more selfishly over the course of their studies. This interpretation, however, must not be valid. A positive correlation of selfish behaviors with study year could be as much due to learning (socialization) as to a systematic dropping out of less selfish economics students (self-selection) as to a third factor, such as the effect of aging in general. Cross-sectional data simply cannot be used for inferring changes within a sample over time. To assess the underlying mechanisms of what is driving the more selfish behavior of economics students longitudinal data sets that include a control group are required.

It is nonetheless possible to speculate that the body of theories on human behavior to which economics students are exposed during their studies can explain their greater skepticism and their more selfish behavior. Economic theories have traditionally been more concerned with the mathematical structure of the decision problem than with the psychology of the individual [53]. Rational choice theory, arguably one of the centerpieces of modern economic theory [54] and the starting point of game theory [55], frames decision-making as a calculative, emotionless weighing of the costs and the benefits of options. Although rational choice theory primarily serves to *describe* decision-making, its application to social situations may cause economics students to *interpret* a situation differently, ultimately leading to different choices [56–58]. For example, Fiske argued that in Western cultures taking calculative approaches to social relations is associated with greater psychological distance [59], less intense moral obligations [60], and more selfishness [61]. Economics students may thus not only learn rational choice theory but also learn to associate it with a specific behavior that they in turn expect from others. Rubinstein, for example, pointed out that students who come to the university “to ‘study economics’ instead become experts in mathematical manipulations” [62]. As a consequence, economics students may be more committed to maximize profits rather than to sympathize with other individuals. Especially through being exposed to game theory in their studies, economics students who participate in game experiments find themselves in social situations for which they have learned the “correct” calculative approach [5]. This does not necessarily mean that economics students are more skeptical and behave more selfishly outside the context of games. Research combining game experiments with field studies would be needed to test how well the choices of economics students—and other students—actually predict their behavior outside the laboratory.

The fact that economics students behave more selfishly than other students is rather critical for experimental practice. Experimental games aim at extrapolating findings from the laboratory to the world beyond [63]. Yet most experimental games are exclusively conducted among university students—especially economics students [64]. Although research suggest that students behave rather similar to non-student population groups [64,65] variation within the student participant pool is frequently neglected, potentially constraining generalization.

## Conclusion

To conclude, this study demonstrated that economics students behaved more selfishly than other students in a third-party punishment game. Analyses of three mechanisms potentially underlying this pattern of results suggest that the more selfish behavior is not due to differences in fairness concerns or notions of fairness, but to the greater skepticism among economics students. This finding sheds new light on the debate about potential links between studying economics and selfish behavior. Selfish behavior is not free from context and can have different motives. In some contexts, economics students behave more selfishly because they expect others to do so.

## Supporting information

S1 Dataset (CSV)

S1 Description Dataset (PDF)

S1 Instructions (PDF)
