# Supplementary material for: The games economists play: Why economics students behave more selfishly than other students
Source: PLoS One. 2017 Sep 5;12(9):e0183814. doi: 10.1371/journal.pone.0183814 (PMC5584942; doi:10.1371/journal.pone.0183814)
Supplement: S1 Description Dataset — (PDF) [file pone.0183814.s002.pdf]

S2 Appendix: Description of the dataset

Variable	Description	Value
AAllocate	Sender's allocation (offer)	0£-12£
BExp	Receiver's expectation of AAllocate (expected offer)	0£-12£
CTreat	The allocation the judge was assigned to	0£-12£
CDecide	The judge's decision to veto	Accept<br>Alter
CChange	The judge's chosen re-allocation (NA if the judge chose not to veto)	0£-12£
Fair	What would be a fair allocation?	0£-12£
Major	The student's study program	Economics<br>Social Sciences<br>Natural Sciences<br>Humanities
Age	The student's age in years	0-99
Gender	The student's gender	Male<br>Female
EstFair	Fairness was mentioned in the comment	1 = yes<br>0 = no
Strategy	The judge was mentioned in the comment	1 = yes<br>0 = no
MaxLanguage	Welfare maximization concerns in the comment	1 = yes<br>0 = no
