# Supplementary material for: The games economists play: Why economics students behave more selfishly than other students
Source: PLoS One. 2017 Sep 5;12(9):e0183814. doi: 10.1371/journal.pone.0183814 (PMC5584942; doi:10.1371/journal.pone.0183814)
Supplement: S1 Instructions — (PDF) [file pone.0183814.s003.pdf]

Game Instructions

THANK YOU FOR PARTICIPATING. This questionnaire will take approximately 7 minutes of your time. Furthermore, you have the chance to obtain real money. Please read the following instructions carefully.

GENERAL INFORMATION. This study is the empirical foundation for a master's thesis at the Department of Social and Developmental Psychology, University of Cambridge. The purpose of this study is to analyse decisions and perceptions in transaction situations. Please note that none of the tasks test your personal intelligence or ability.

CONSENT. This study has received ethical approval from the Departmental Research Committee. Participation is voluntary and you may refuse to answer certain questions. You may withdraw from the study at any time without penalty. This does not waive your legal rights. Results may be presented at conferences and published in journals. Results are presented in terms of groups of individuals. Any data presented would be totally anonymous.

At the end of this study, you may state your e-mail address. This is the only confidential data recorded. It will not be forwarded, and no third party will have access. It is retained purely to inform you of the study's outcome. Your e-mail address and all other data are handled in accordance with the University's Data Protection Act, 1998 (for further information, please see http://www.admin.cam.ac.uk/univ/information/dpa/).

PROCEDURE & PAYMENT. The study is in two parts. In Part One, participants make decisions in a transaction situation. This involves real money transfers. Twenty-one participants will be randomly chosen and paid according to these transactions. In end-February, randomly selected participants are informed and paid. In Part Two, participants are asked about their habits and personal background. If you have any further questions, please contact the conducting researcher.

TRANSACTION SITUATION. This study comprises three roles: A, B, and C. You are asked to make decisions in each one sequentially. At the end of the study, you are randomly assigned to one role. All roles are assigned to real people with whom you will interact. The interaction does not repeat itself.

STAGE ONE. In Stage One, role A is the sole decision-maker, B is passive and C simply observes the interaction. Role A gets an endowment of £12. Passive role B gets no endowment. Active role A must decide how much of the £12 is assigned to passive role B. Role A can transfer any figure in pound sterling between 0 and 12. If, for example, role A grants role B £5, role A’s income at the end of stage one will amount to £7, and role B’s income will be £5. If role A grants role B £0, role A’s income will be £12, and role B’s will be £0.

STAGE TWO. In Stage Two, role C is the sole decision-maker. Roles A and B are entirely passive. Role C has two options. They can either accept or alter role A’s decision in Stage One. If role C decides to accept role A’s decision, A and B are paid in accordance with Stage One. Role C receives £7 as an endowment and the transaction is complete. If role C decides to alter role A’s decision, role C’s endowment is limited to £5. However, role C may then proceed to reallocate a new figure in pound sterling between 0 and 12 to role B. If, for example, role A decided to allocate £0 to role B in Stage One and role C disagrees, role C might alter the allocation to £9. In consequence, role A’s income would be £3.

You are asked to make the decisions in each of these roles.

THIS IS STAGE ONE. You are role B. How much of the £12 do you expect role A will decide to allocate to you?

[ ] £0 [ ] £1 [ ] £2 [ ] £3 [ ] £4 [ ] £5 [ ] £6 [ ] £7 [ ] £8 [ ] £9 [ ] £10 [ ] £11 [ ] £12

THIS IS STILL STAGE ONE. You are now role A. How much of the £12 do you wish to allocate to role B?

[ ] £0 [ ] £1 [ ] £2 [ ] £3 [ ] £4 [ ] £5 [ ] £6 [ ] £7 [ ] £8 [ ] £9 [ ] £10 [ ] £11 [ ] £12

In two to four sentences, please explain the reason behind your decision.

[ ]

THIS IS STAGE TWO. You are role C. Role A decided to grant role B £[0–6].

[ ] I accept the allocation. Thus, I get £7; role B gets £[0–6]; and role A will receive £[6–0].

[ ] I alter the allocation. My endowment is limited to £5, and I reallocate the money to role B.

THIS IS STILL STAGE TWO. You decided to alter role A's decision. Please state how much you want role B to obtain. Role A will receive the remainder.

[ ] £0 [ ] £1 [ ] £2 [ ] £3 [ ] £4 [ ] £5 [ ] £6 [ ] £7 [ ] £8 [ ] £9 [ ] £10 [ ] £11 [ ] £12

THE TRANSACTION HAS NOW ENDED. In the following, we ask you questions about your perception and personal background.

PERCEPTION. What would be a fair allocation of the £12 to role B?

[ ] £0 [ ] £1 [ ] £2 [ ] £3 [ ] £4 [ ] £5 [ ] £6 [ ] £7 [ ] £8 [ ] £9 [ ] £10 [ ] £11 [ ] £12 [ ] don't know
